# Supplementary material for: Molecular architecture of the N‐type ATPase rotor ring from Burkholderia pseudomallei
Source: EMBO Rep. 2017 Mar 10;18(4):526–35. doi: 10.15252/embr.201643374 (PMC5376962; doi:10.15252/embr.201643374)
Supplement: Supplementary file 1 — Expanded View Figures PDF [file EMBR-18-526-s001.pdf]

# Expanded View Figures

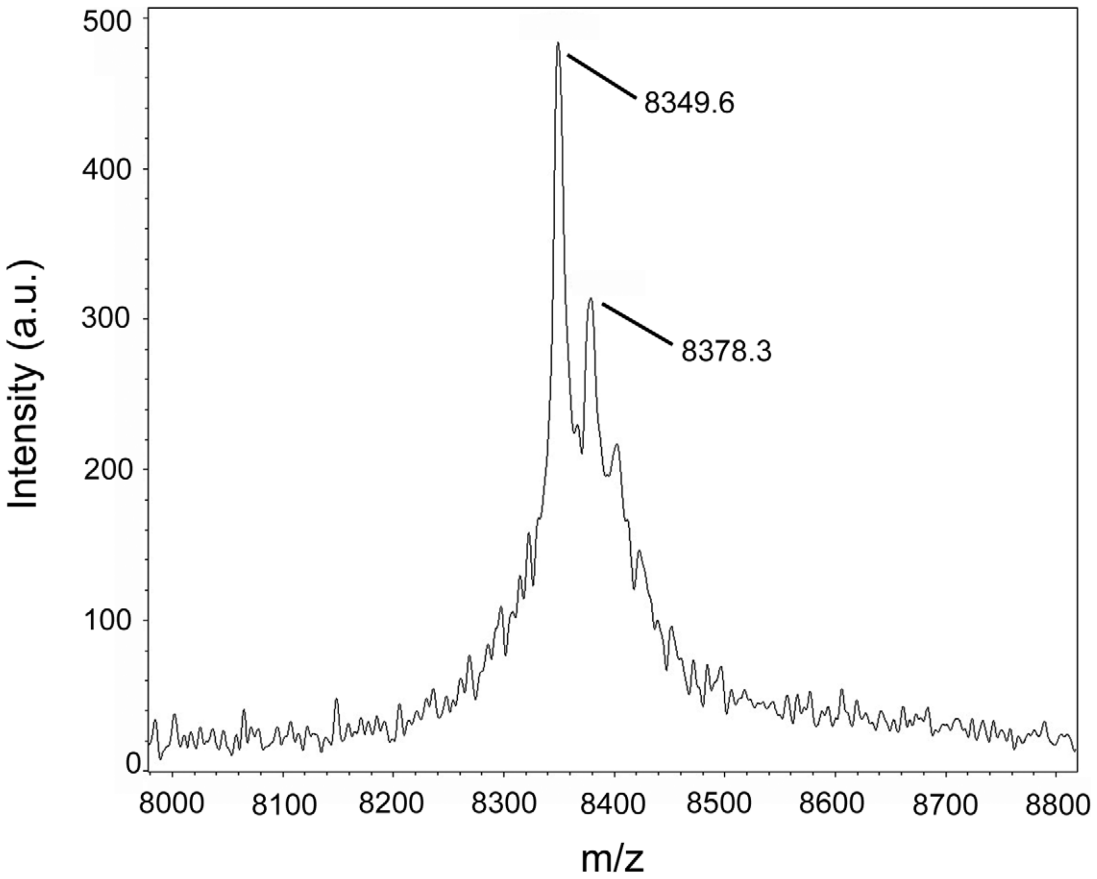


**Figure EV1. MALDI mass spectrum of purified *Burkholderia pseudomallei* c-subunit.**
Peaks were identified at 8,349.6 m/z and 8,378.3 m/z, corresponding to the molecular mass of unformylated (predicted 8,349.96 Da) or formylated (predicted 8,377.96 Da) *B. pseudomallei* c-subunit.

© 2017 The Authors

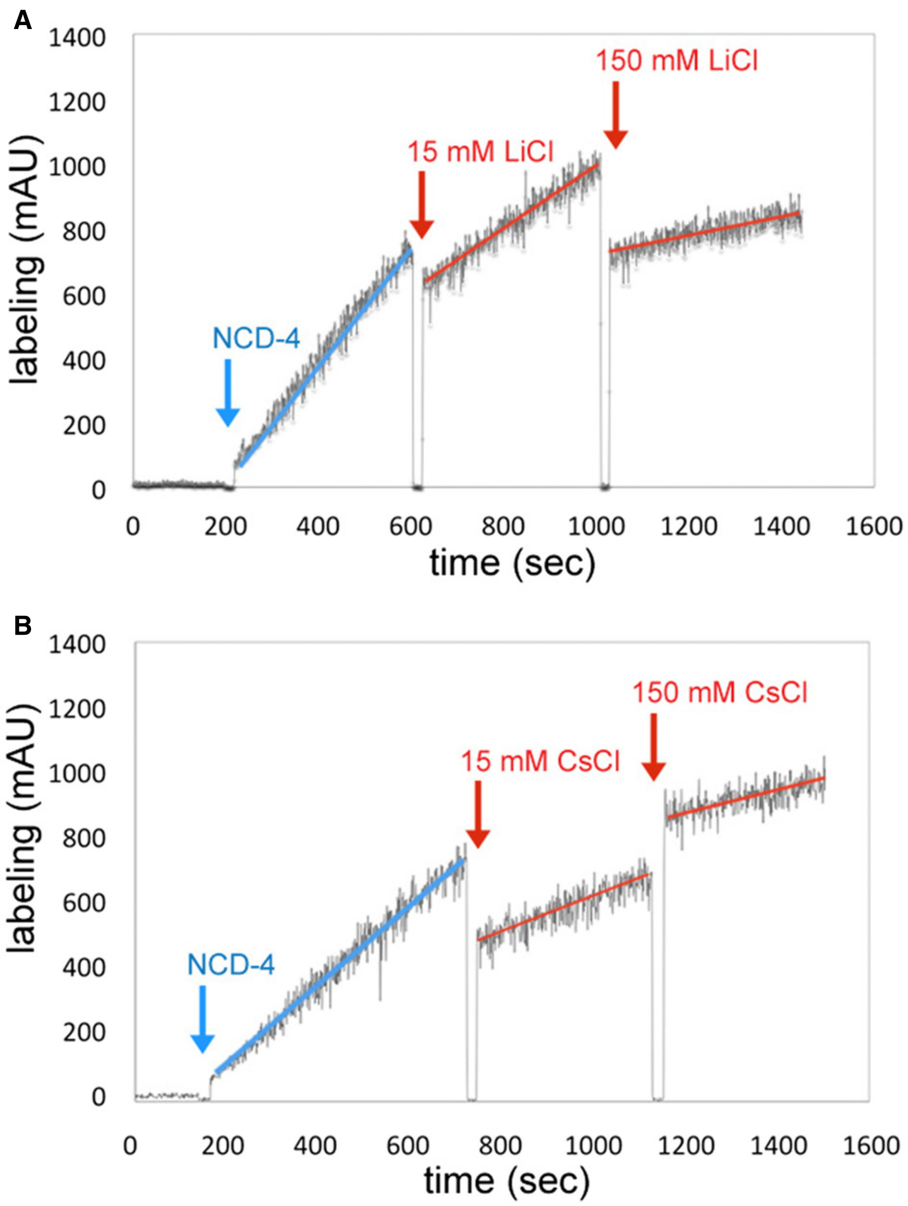


**Figure EV2. Effect of $Li^+$ and $Cs^+$ on the kinetics of the NCD-4 modification of the *Burkholderia pseudomallei* N-type ATPase c-ring.**

100 μM NCD-4 was added to the purified c-ring in 1.0% DDM and MES buffer, pH 6.0. The trace shows the continuous increase of fluorescence upon NCD-4 binding to Glu61 of the c-ring.

A Addition of 15 or 150 mM LiCl resulted in a drop in labeling efficiency to 55 or 17%.

B Addition of 15 or 150 mM CsCl reduced the labeling efficiency to 44 or 24%, illustrating the effect of salt in the buffer [33] and the overall dilution of the reaction volume. Results of typical experiments are shown. Each experiment was repeated three times.

 © 2017 The Authors

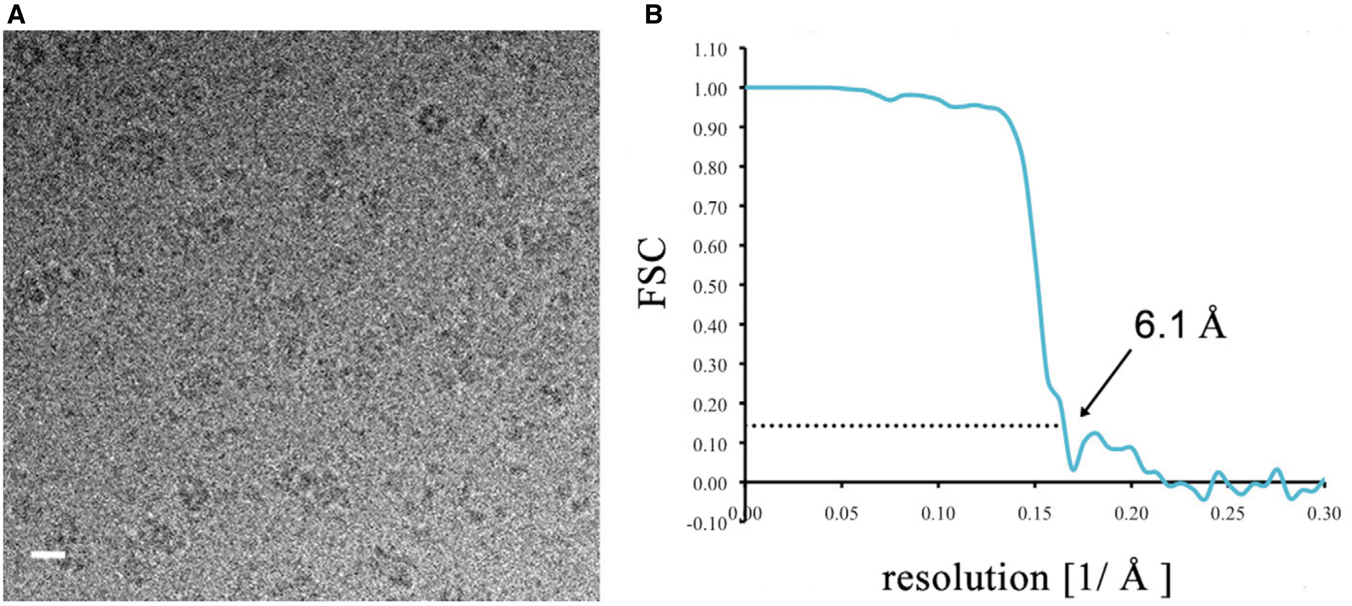


**Figure EV3. Electron cryo-microscopy of the *Burkholderia pseudomallei* N-type ATPase rotor ring.**

A Representative electron micrograph of rotor rings in vitreous buffer (scale bar, 10 nm).

B Gold-standard FSC curve [59] for the density map of the rotor ring in LDAO. The resolution at 0.143 FSC was 6.1 Å (arrow).

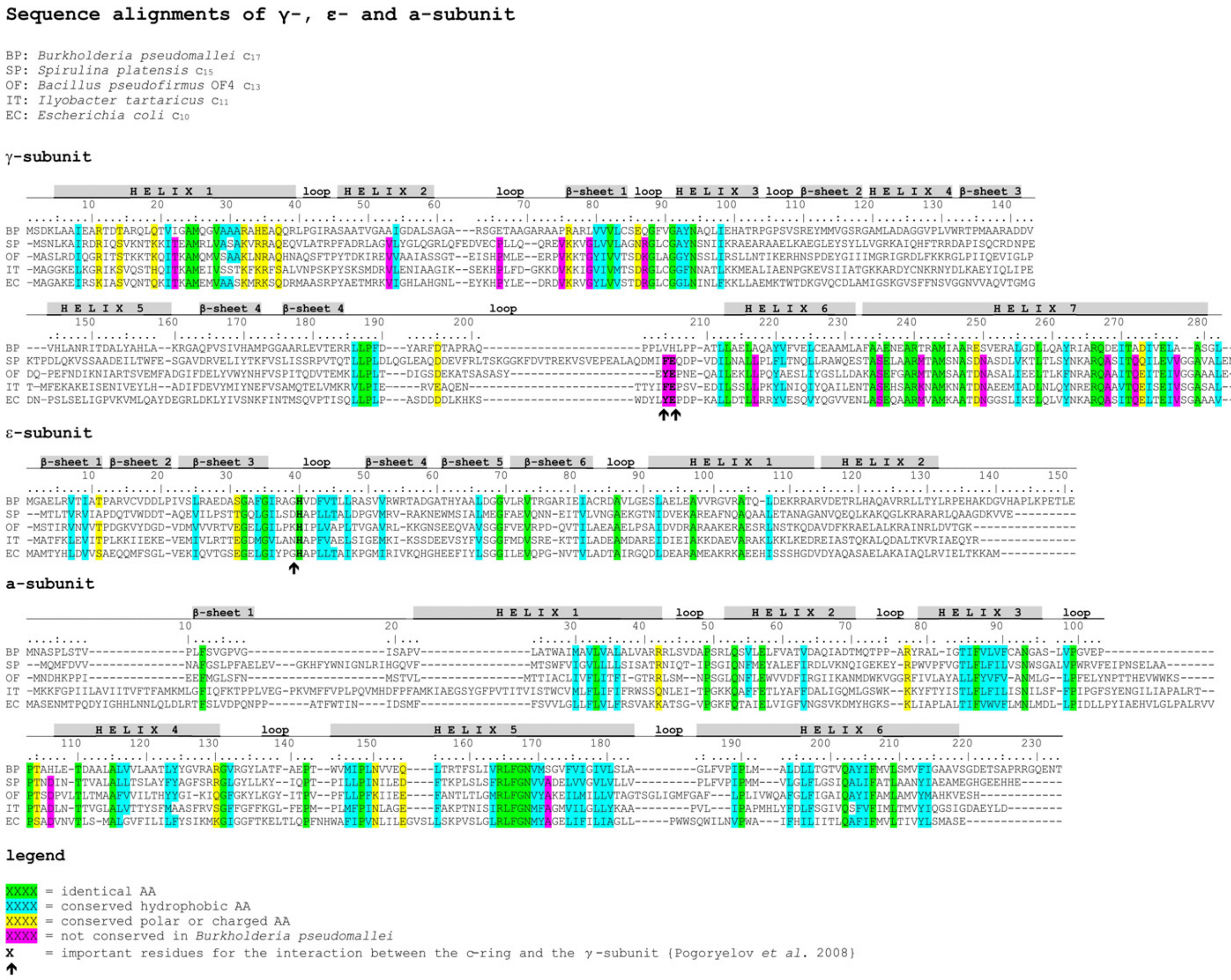


**Figure EV4. Subunits adjacent to the c-ring of N-type ATPase.**

Alignment of F-type and N-type *atpG*, *atpC*, and *atpB*, comparing sequences of the predicted γ-, ε-, and a-subunits of *Burkholderia pseudomallei* (BC), *Spirulina platensis* (SP), *Bacillus pseudofirmus* OF4 (OF), *Ilyobacter tartaricus* (IT), and *Escherichia coli* (EC) having a $c_{17}$, $c_{15}$, $c_{13}$, $c_{11}$, and $c_{10}$ ring. Differences were observed in the γ-subunit of *B. pseudomallei*.

 © 2017 The Authors

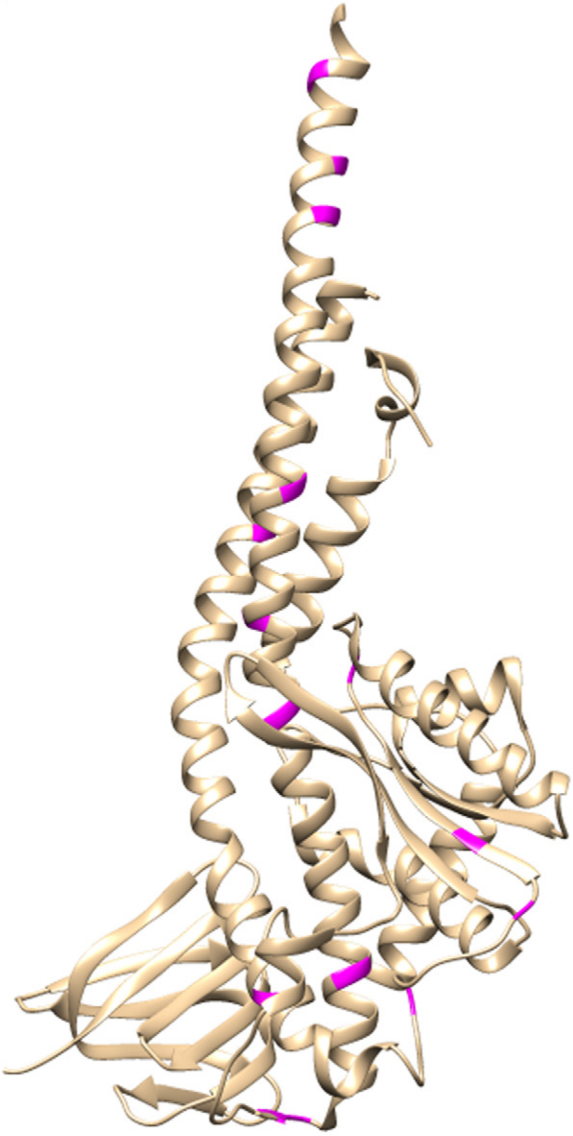


**Figure EV5. Cartoon model of the *Escherichia coli* γ-subunit.**
The structure from Cingolani & Duncan [60] was used. The positions of different residues marked in magenta in Fig EV4 are highlighted in the *E. coli* structure. Glu208 and Tyr207, which are exchanged to Val and His in *Burkholderia pseudomallei* interact with the c-ring [61].
