# Supplementary material for: Molecular architecture of the N‐type ATPase rotor ring from Burkholderia pseudomallei
Source: EMBO Rep. 2017 Mar 10;18(4):526–35. doi: 10.15252/embr.201643374 (PMC5376962; doi:10.15252/embr.201643374)
Supplement: Supplementary file 2 — Table EV1 [file EMBR-18-526-s002.docx]

**Table EV1. Quantification of NCD-4 labeling efficiency of the *B. pseudofirmus* c_17_ ring.**

|  | **NaCl, pH 6**  **(mM)** | | | **LiCl), pH 6**  **(mM)** | | | **CsCl, pH 6**  **(mM)** | | | **pH 9 shift** | |
| --- | --- | --- | --- | --- | --- | --- | --- | --- | --- | --- | --- |
|  | **0** | **15** | **150** | **0** | **15** | **150** | **0** | **15** | **150** | **pH 6** | **pH 9** |
| **labeling^*^** | 100% | 40% | 27% | 100% | 55% | 17% | 100% | 44% | 24% | 100% | 1% |
| **Time (min)** | 0 | 6.5 | 13 | 0 | 6.5 | 13 | 0 | 6.5 | 13 | 0 | (6.5)^**^ |

^*^the initial labeling rate was taken as 100%.

^**^immediately after addition
