# Supplementary material for: Molecular architecture of the N‐type ATPase rotor ring from Burkholderia pseudomallei
Source: EMBO Rep. 2017 Mar 10;18(4):526–35. doi: 10.15252/embr.201643374 (PMC5376962; doi:10.15252/embr.201643374)
Supplement: Supplementary file 3 — Table EV2 [file EMBR-18-526-s003.docx]

**Table EV2. Densities and micelle sizes of different solubilizing agents, vitrified ice and protein.**

| **Amphipathic molecule** | **Density [g/ml]** | **Micelle size [kDa]** | **Reference** |
| --- | --- | --- | --- |
|  |  |  |  |
| **Protein** | ~1.36 | - | (Fischer, Polikarpov et al., 2004, Kühlbrandt, 1982) |
| **Amphipol A8‑35** | 1.3-1.19 | 70 kDa | (Tribet, Audebert et al., 1996) |
| **DDM** | 1.19 | 72 kDa | (Timmins, Leonhard et al., 1988) |
| **C_12_E_8_** | 1.04 | 65 kDa | (le Maire, Champeil et al., 2000) |
| **Amorphous ice** | 0.94 | - | (Mishima, Calvert et al., 1985) |
| **LDAO** | 0.88 | 17 kDa | (Timmins et al., 1988) |

Fischer H, Polikarpov I, Craievich AF (2004) Average protein density is a molecular-weight-dependent function. Protein Sci 13: 2825 – 2828 15

le Maire M, Champeil P, Moller JV (2000) Interaction of membrane proteins and lipids with solubilizing detergents. Biochim Biophys Acta 1508: 86 – 111 16

Mishima O, Calvert LD, Whalley E (1985) An apparently first-order transition between two amorphous phases of ice induced by pressure. Nature 314: 76 – 78 17

Timmins PA, Leonhard M, Weltzien HU, Wacker T, Welte W (1988) A physical characterization of some detergents of potential use for membrane protein crystallization. FEBS Lett 238: 361 – 368 18

Tribet C, Audebert R, Popot JL (1996) Amphipols: polymers that keep membrane proteins soluble in aqueous solutions. Proc Natl Acad Sci USA 93: 15047 – 15050
