# Supplementary material for: Molecular architecture of the N‐type ATPase rotor ring from Burkholderia pseudomallei
Source: EMBO Rep. 2017 Mar 10;18(4):526–35. doi: 10.15252/embr.201643374 (PMC5376962; doi:10.15252/embr.201643374)
Supplement: Supplementary file 4 — Movie EV1 [file EMBR-18-526-s004.zip › Schulz_etal_Movie_EV1_legend.docx]

**Movie EV1. Structural architecture of the c-ring of the N-type ATPase from *Burkholderia pseudomallei* determined by electron cryo‑microscopy.** The cryo-EM map resolves the H^+^ binding membrane rotor at 6.1 Å resolution and reveals a heptadecameric oligomer with a molecular mass of ~141 kDa. Each c-subunit consists of an α-helical hairpin (shown as cartoon model in rainbow colors) and contains one proton binding site. The ion-to-ATP ratio that is defined by the c_17_ ring in this N-type ATPase is 5.7 (3 ATP per 17 protons).
